# Baroreflex activity through the analysis of the cardio-respiratory variability influence over blood pressure in cardiomyopathy patients

**DOI:** 10.3389/fphys.2023.1184293

**Published:** 2023-08-10

**Authors:** Javier Rodriguez, Steffen Schulz, Andreas Voss, Sergio Herrera, Salvador Benito, Beatriz F. Giraldo

**Affiliations:** ^1^ Automatic Control Department (ESAII), Barcelona East School of Engineering (EEBE), Universitat Politècnica de Catalunya (UPC), Barcelona, Spain; ^2^ Institute for Bioengineering of Catalonia (IBEC), The Barcelona Institute of Science and Technology, Barcelona, Spain; ^3^ Institute of Innovative Health Technologies, Jena, Germany; ^4^ Hospital de la Santa Creu i Sant Pau, Barcelona, Spain; ^5^ CIBER de Bioengenieria, Biomateriales y Nanomedicina (CIBER-BBN), Barcelona, Spain

**Keywords:** baroreflex activity, cardio-respiratory variability, blood pressure variability, morphology-relative change, ischemic-dilated cardiomyopathy

## Abstract

A large portion of the elderly population are affected by cardiovascular diseases. Early prognosis of cardiomyopathies remains a challenge. The aim of this study was to classify cardiomyopathy patients by their etiology based on significant indexes extracted from the characterization of the baroreflex mechanism in function of the influence of the cardio-respiratory activity over the blood pressure. Forty-one cardiomyopathy patients (CMP) classified as ischemic (ICM—24 patients) and dilated (DCM—17 patients) were considered. In addition, thirty-nine control (CON) subjects were used as reference. The beat-to-beat (BBI) time series, from the electrocardiographic (ECG) signal, the systolic (SBP), and diastolic (DBP) time series, from the blood pressure signal (BP), and the respiratory time (TT), from the respiratory flow (RF) signal, were extracted. The three-dimensional representation of the cardiorespiratory and vascular activities was characterized geometrically, by fitting a polygon that contains 95% of data, and by statistical descriptive indices. DCM patients presented specific patterns in the respiratory response to decreasing blood pressure activity. ICM patients presented more stable cardiorespiratory activity in comparison with DCM patients. In general, CMP shown limited ability to regulate changes in blood pressure. In addition, patients also shown a limited ability of their cardiac and respiratory systems response to regulate incremental changes of the vascular variability and a lower heart rate variability. The best classifiers were used to build support vector machine models. The optimal model to classify ICM *versus* DCM patients achieved 92.7% accuracy, 94.1% sensitivity, and 91.7% specificity. When comparing CMP patients and CON subjects, the best model achieved 86.2% accuracy, 82.9% sensitivity, and 89.7% specificity. When comparing ICM patients and CON subjects, the best model achieved 88.9% accuracy, 87.5% sensitivity, and 89.7% specificity. When comparing DCM patients and CON subjects, the best model achieved 87.5% accuracy, 76.5% sensitivity, and 92.3% specificity. In conclusion, this study introduced a new method for the classification of patients by their etiology based on new indices from the analysis of the baroreflex mechanism.

## 1 Introduction

One of the most relevant challenges in biomedical research is the analysis of system response variability. This is due not only because a system’s response variability represents the description of the patient’s state, but also because it can mask comorbidities. Diseases like cardiomyopathies, which affect a large segment of the elderly population, are of particular interest, as they constitute one of the most common causes of death. The clinical diagnosis of these patients, especially the ones with multiple comorbidities, are still challenging.

These pathologies may originate in an ischemic (ICM) or dilated (DCM) process of the heart. The reaction to this process, however, may be related to the variability of the cardiorespiratory and cardiovascular system response, which is associated with the baroreflex mechanism. Therefore, these systems can help in differentiating between these cardiomyopathies, which can lead to an improved and earlier diagnosis for these patients (Medina et al., 1985; Sawada MD et al., 1992). For instance, patients that suffers from ischemic related cardiomyopathies develops a worse prognosis and are candidates for a more specialized therapy. Some authors suggest that there is a high prevalence of etiologic misclassification in advanced heart failure (Mehra and Uber, 2013). According to their research, at least 20% of the patients were misclassified.

Despite the symptomatic similarities between ischemic and dilated cardiomyopathies, they differ in etiology. For instance, ischemic cardiomyopathy (ICM) is related to coronary artery disease, and is characterized by myocyte loss, a compensatory tissue hypertrophy, and ventricular scarring (Beltrami et al., 1994). ICM patients may be treated according to their heart damage with medications such as nitrates, beta blockers, angiotensin-converting enzyme inhibitors, among others. In more critical cases, the treatment can include procedures like angioplasty or coronary artery bypass. On the other hand, dilated cardiomyopathy (DCM) is characterized by the enlargement and weakening of the left ventricle and contractile dysfunction (McNally Elizabeth and Mestroni, 2017). DCM may be associated with an increased risk of severe arrhythmia. Treatment of DCM patients could include medications like angiotensin II beta blockers, diuretics, digoxin, among others. Additionally, in some cases is necessary to implant devices such as the implantable cardioverter-defibrillator, left ventricular assist devices or a biventricular peacemaker. Considering the differences mentioned, new knowledge that could help improving the diagnosis of these type of patients is desired.

Several linear and non-linear techniques based on the analysis of biomedical signals have been explored in earlier studies (Davos et al., 2002; Stein et al., 2005; Voss et al., 2010). Deep learning methods have been developed to predict ventricular fibrillation episodes in cardiomyopathy patients (Tseng and Tseng, 2020), and to predict heart failure in patients with different levels of left ventricular ejection fraction (Alkhodari1 et al., 2021). Machine learning techniques have been used to predict cardiac-related prognosis in different types of patients (Guisen et al., 2021), for the prediction of complications after cardiovascular surgery (Jiang1 et al., 2021), and for the prediction of the progression of heart failure in hypertrophic cardiomyopathy (Fahmy et al., 2021). Support vector machine-based algorithms have been explored for the detection of cardiac disorders (Wang and Zheng, 2009; Borkar and Annadate, 2018). Differences in the cardiovascular autonomic regulation of ischemic and dilated cardiomyopathy patients have been hypothesized (Freeman et al., 2006). The calcium release during the excitation-contraction coupling of the heart was investigated in both ischemic and dilated cardiomyopathy patients (Brillantes et al., 1992). The authors found that sarcoplasmic reticulum calcium release channel levels were 28% lower in ICM patients, suggesting that ischemic hearts exhibit diminished calcium release during excitation-contraction. This pathological behavior could be caused by the abnormal calcium processing of myopathic cardiac muscle in ischemic heart disease.

Other authors measured the content and activities of components of the β-adrenergic receptor-G protein-adenylate cyclase complex and adrenergic neurotransmitter levels in the left and right ventricular myocardium of patients with ischemic and dilated cardiomyopathy. They revealed that there are differences between the regulatory β-receptor-effector related mechanisms in ICM vs. DCM patients (Bristow et al., 1991). ICM patients were characterized by less β-adrenergic receptor downregulation in both ventricles, a decreased coupling of β-adrenergic receptors mediating a contractile response in right ventricular tissue, and decreased coupling of β-adrenergic receptors mediating adenylate cyclase stimulation in the left ventricular tissue.

Another study compared the myocardial tissue from ischemic and dilated cardiomyopathy patients (Böhm et al., 1990). The authors found a reduction in positive inotropic effects, leading to weaker contractions in DCM patients compared to ICM patients. This result suggests that increased levels of heterotrimeric G-proteins and reduced basal adenylate cyclase activity give rise to the abnormal regulation of contractility in dilated hearts. Other authors have used both neural networks and fuzzy methods to classify different heart diseases, including ischemic and dilated cardiomyopathies (Acharya et al., 2004).

Analyzing the interactions between the cardiovascular and respiratory systems could provide new insights into these cardiomyopathies and contribute to the more accurate diagnosis of these diseases. The coupled influence between the respiratory and cardiovascular systems under pathological conditions have been explored before (Hernando et al., 2013). In our previous work, we proposed analyzing the cardiovascular activity using coupling analysis (Rodriguez et al., 2019). In this work, we introduce cardiorespiratory interaction as a means by which to analyze the behavior of the systems associated with ICM and DCM cardiomyopathies. We proposed a three-dimensional analysis that considered the relationship between the cardiac, respiratory, and vascular systems. Based on vascular activity as the input and output of the baroreflex response, we evaluated the variability of these interactions. The aim of this study was to analyze the suitability of cardiorespiratory and vascular interactions for the classification of ICM and DCM patients. However, we also characterized these interactions through features extracted from electrocardiography (ECG), respiratory flow and blood pressure signals.

## 2 Data acquisition

Noninvasive electrocardiographic (ECG), blood pressure (BP) and respiratory flow (RF) signals from 41 cardiomyopathy patients were registered at the Santa Creu i Sant Pau Hospital in Barcelona, Spain. All records were performed according to a protocol, previously approved by the Hospital ethics committee. Forty-one cardiomyopathy patients were used to explore the method. The patients were characterized by New York Heart Association functional classification (NYHA) ≥ 2 and were diagnosed with either ischemic cardiomyopathy (ICM—24) or dilated cardiomyopathy (DCM—17). In addition, thirty-nine healthy subjects (12 male, 27 female; aged 27 ± 7 years) were used as the control group (CON), maintaining the anamnestic features of the cases. The clinical information is summarized in Table 1. The recordings were acquired with the Portapres-system and the Porti 16-biosignal amplifier, for 15 min, at a sample frequency of 1600 Hz, with the patient in supine position (Rodriguez et al., 2019).

**TABLE 1:** Clinical parameters (mean and standard deviation).

	ICM	DCM
Patients	24	17
Age [years]	65.4 ± 10.53	61.54 ± 12.27
Weight [kg]	78.14 ± 14.22	77.40 ± 16.47
BMI [kg/m^2^]	27.55 ± 3.89	27.76 ± 5.77
NYHA	2.07 ± 0.28	2 ± 0.57
LVDD [mm]	60.83 ± 8.78	62.75 ± 4.27
AD [mm]	46.22 ± 7.67	44.62 ± 3.47
ProBNP	881.78 ± 1424.4	441.8 ± 1535.7
LVEF [%]	33.4 ± 11.49	33.47 ± 6.68

ICM, ischemic cardiomyopathy; DCM, dilated cardiomyopathy; BMI, body mass index; NYHA, new york heart association functional classification; LVDD, left ventricular diastolic dysfunction; AD, auricular diameter; ProBNP, brain natriuretic peptide; LVEF, left ventricular ejection fraction.

## 3 Signal processing

The ECG, BP and RF signals linear trend were removed, and in-house preprocessing tools were used to reduce noise, artifacts and spikes. Any sample with a value equal to or greater than 3 times the standard deviation of the series was considered an outlier. All outliers were eliminated. The beat-to-beat interval time series (BBI) were extracted from the ECG signal using an in-house algorithm that detects the R peaks and calculate the time between peaks, milliseconds. The systolic (SBP) and diastolic (DBP) blood pressure time series were calculated as the maximum and minimum values of the BP in each heartbeat, in mmHg. The total respiratory cycle time series (TT) was assessed as the time between two successive breath periods, in seconds. Thereafter, all-time series were inspected and edited, if necessary.

In order to analyze the cardiac, respiratory and vascular interactions, the time series were synchronized using linear interpolation method with a sample frequency of 1 Hz. To properly observe and analyze changes in the respiratory activity, the time series were decimated to 0.25 Hz.

## 4 Methodology

To analyze the interaction between the cardiac, respiratory and vascular systems, we took the difference between two consecutive events $\left(∆X\right)$ as a new time series values, represented by,

$$∆X=X_{n+1}-X_{n}\forall n=1,\ldots ,N$$ (1)

being X either BBI , SBP , DBP or TT time series, and N total number of time series value.

To determine vascular activity, we defined a threshold associated with the increase, decrease or absence of change of the SBP or DBP variability, defined by:

$$ThX_{v}=\frac{\alpha \left(\left|\max\left(∆X_{v}\right)\right|-\left|\min\left(∆X_{v}\right)\right|\right)}{2}$$ (2)

where $∆X_{v}$ is either ∆SBP or ∆DBP time series, and α is a factor that defines the percentage of the boundary value at which blood pressure values are considered as “no change”. The dataset was normalized (zero mean and unit variance) and centered to zero. In this study, we defined α = 15%. All parameters were normalized.

To analyze the variability of the vascular system, three new sub-spaces were defined in accordance with the $ThX_{v}$ threshold: “up” referred to increasing values, “no change” referred to values between the negative and positive threshold, and “down” referred to decreasing values. Thus, the values of each $∆X_{v}$ are classified as:

$$\begin{array}{c} -Up\left(u\right):∆X_{v}>ThX_{v}\\ -No\;change:\left(nc\right):-ThX_{v}\leq ∆X_{v}\leq ThX_{v}\\ -Down\left(d\right):∆X_{v}<-ThX_{v} \end{array}$$

**FIGURE 1:**
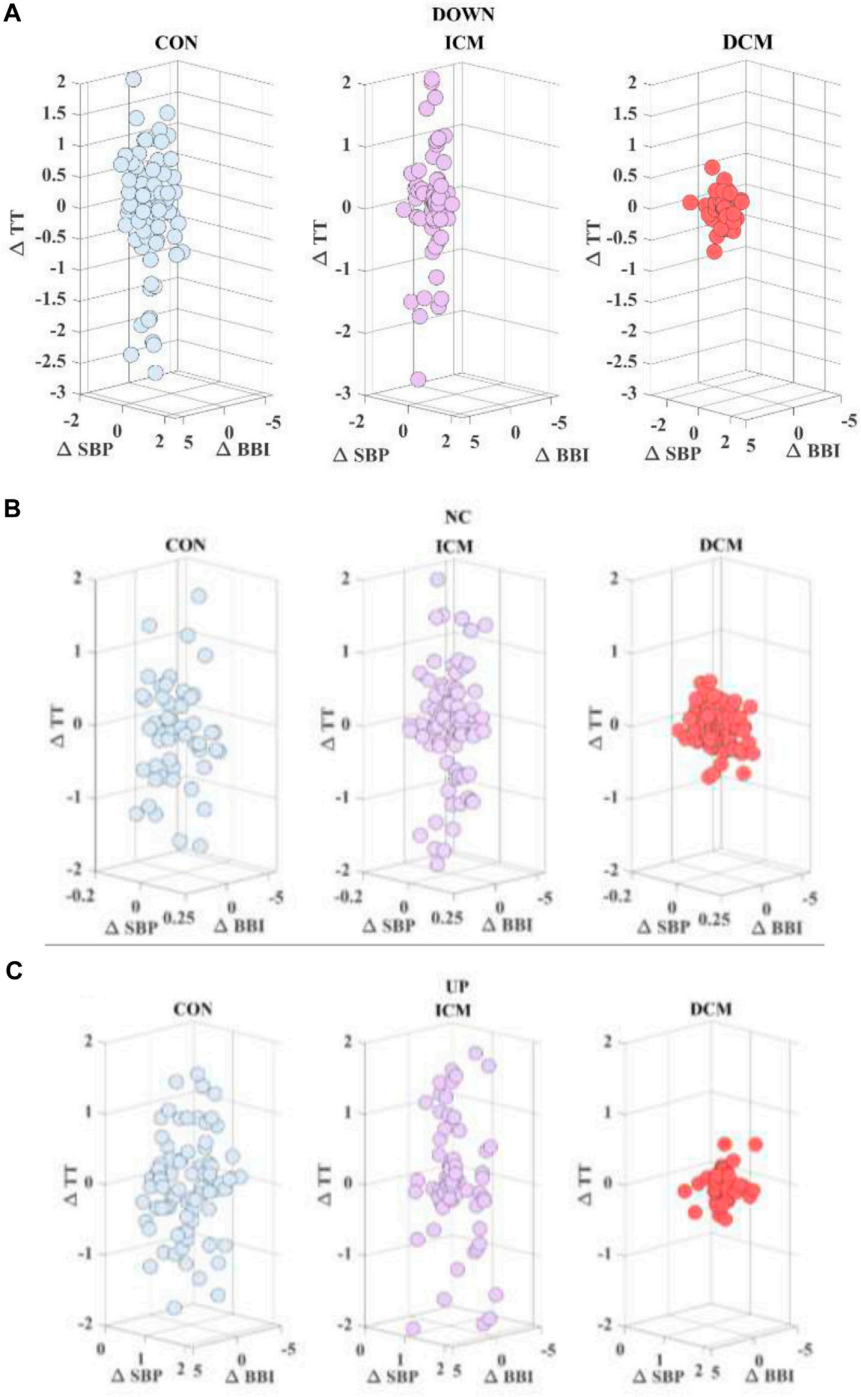
Cardiac (∆BBI) *vs*. respiratory (∆TT) time series considering (∆SBP) **(A)** down, **(B)** no change, and **(C)** up activity, for a CON subject, an ICM patient and a DCM patient.

To evaluate the variability of cardiorespiratory activity associated with vascular behavior, a three-dimensional representation was generated for each sub-space: “up”, “no change” and “down”. This representation presents the relationship between changes in the ∆BBI , ∆TT , ∆SBP and ∆DBP time series (arbitrary units). Figure 1 is an example of the representation of these relationships for a subject from the control group (CON), an ischemic patient (ICM), and a dilated patient (DCM) based on ∆SBP .

Several parameters were extracted from this three-dimensional representation in order to characterize their interactions. For each subject/patient scatterplot, a polygon that represents the projection of the cardiac and respiratory activity based on vascular behavior ( ∆SBP and ∆DBP ) was fitted. The vertices of the polygon were defined including 95% of each set of values. To characterize the geometry of each polygon, its area ( $∆{XY}_{A}$ ) and centroid ( $C_{x}$ , $C_{y}$ ) were defined by:

$$\begin{array}{c} ∆{XY}_{A}=\frac{1}{2}\sum_{i=0}^{n-1}\left(V_{X_{i}}V_{Y_{i+1}}-V_{X_{i+1}}V_{Y_{i}}\right)\\ C_{x}=\frac{1}{6A}\sum_{i=0}^{n-1}\left(V_{X_{i}}+V_{X_{i+1}}\right)\left(V_{X_{i}}V_{Y_{i+1}}-V_{X_{i+1}}V_{Y_{i}}\right)\\ C_{y}=\frac{1}{6A}\sum_{i=0}^{n-1}\left(V_{Y_{i}}+V_{Y_{i+1}}\right)\left(V_{X_{i}}V_{Y_{i+1}}-V_{X_{i+1}}V_{Y_{i}}\right) \end{array}$$ (3)

where $V_{X_{i}}$ and $V_{Y_{i}}$ are coordinates of the *i*-th vertex, and n is the total number of vertices. The area was normalized in accordance with the number of vertices ( $∆{XY}_{A_{v}}$ ) and samples ( $∆{XY}_{A_{n}}$ ). In addition, the mean distance between each sample and centroid was measured ( $∆{XY}_{dc}$ ), and the probability of occurrence of samples for each triangle of the polygon ( $∆X_{p}$ ) was calculated.

**FIGURE 2:**
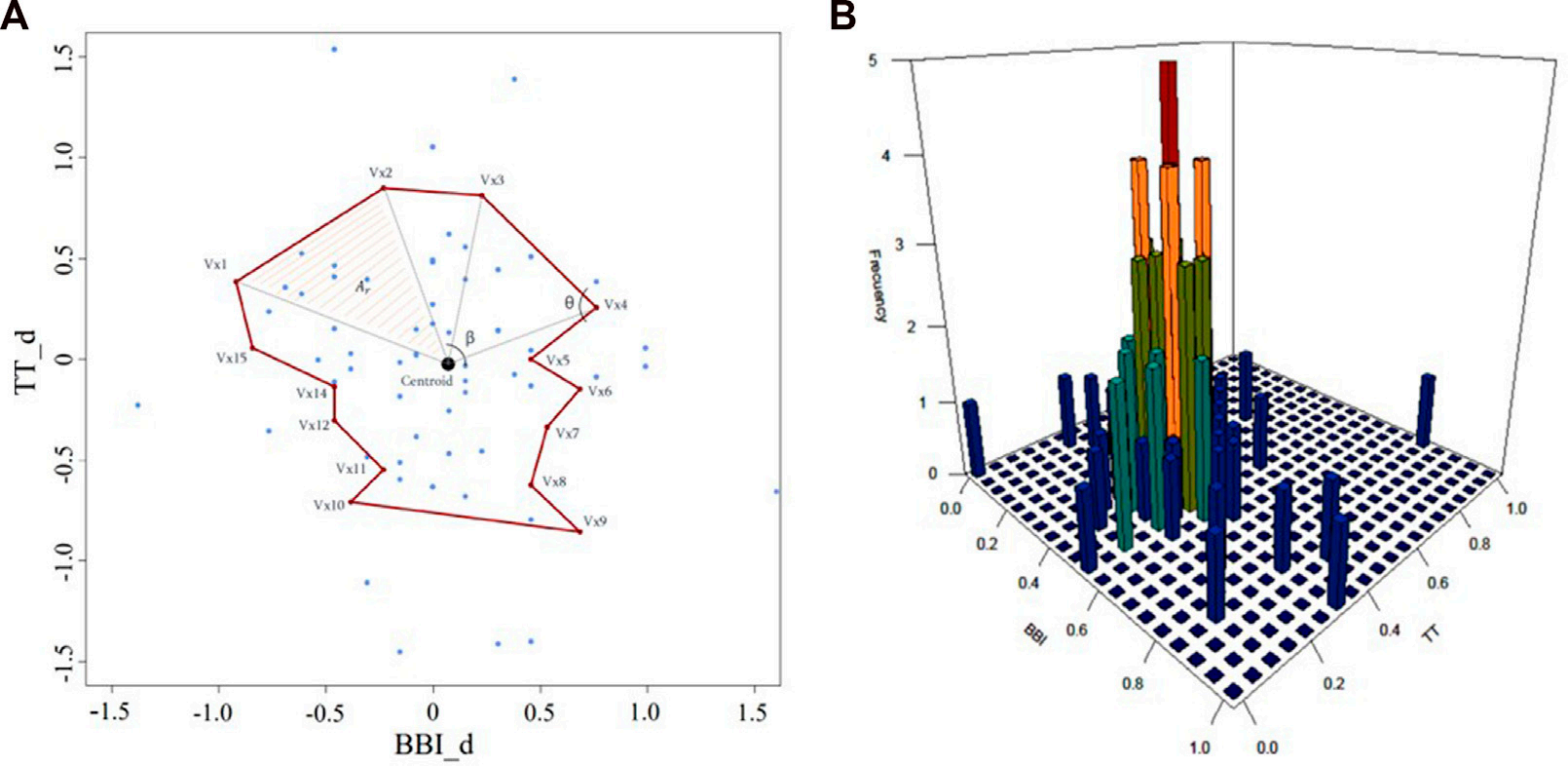
Scatterplot of cardiorespiratory activity for decreasing systolic vascular activity in a CON subject. **(A)** morphological characterization including $V_{x1}-V_{x15}$ : vertices of the polygon; $A_{r}$ : area of the triangle formed by vertices $V_{x1}$ and $V_{x2}$ , and the centroid; θ and β: angles formed on the $V_{x4}$ vertex and the centroid, respectively, and **(B)** statistical characterization.

The morphology of the polygon was also analyzed using all the triangles that form it. First, the mean area of all triangles ( $∆{XY}_{A_{r}}$ ) and the probability of occurrence of the samples ( $∆{XY}_{\mathrm{pr}}$ ) were calculated, and then the mean angles formed were calculated: one in the centroid with two consecutive vertices ( $∆{XY}_{\beta }$ ) and a second in the vertex with the centroid ( $∆{XY}_{ϴ}$ ). Figure 2 is an example of the morphological characterization of a scatterplot for cardiorespiratory activity, when systolic vascular activity decreased, illustrated for a CON subject. In addition, for the ∆BBI and ∆TT time series, statistical parameters like mean ( $∆X_{m}$ ), standard deviation ( $∆X_{sd}$ ), kurtosis ( $∆X_{K}$ ), skewness ( $∆X_{Sk}$ ), interquartile range ( $∆X_{IQR}$ ), and coefficient of variation ( $∆X_{CV}$ ) were obtained for each sub-space (up, no-change, and down). Table 2 shows a summary of the indices considered.

**TABLE 2:** Index description.

Index	Description
$∆X_{m}\_z$	∆X time series mean value
∆sd_z	∆X time series standard deviation
$∆X_{K}\_z$	∆X time series standard Kurtosis
$∆X_{Sk}\_z$	∆X time series Skewness
$∆X_{IQR}\_z$	∆X time series coefficient of variation
$∆X_{CV}\_z$	∆X time series interquartile range
$∆X_{p}\_z$	∆X time series probability of occurrence
$∆X_{d}\_z$	∆X time series mean distance
$∆{XY}_{A}\_z$	Area of the fitting polygon for the X and Y time series
$∆{XY}_{A_{n}}\_z$	Area of the fitting polygon normalized by the number of points
$∆{XY}_{A_{v}}\_z$	Area of the fitting polygon normalized by the number of vertices
$∆{XY}_{n}\_z$	Number of vertices of the fitted polygon
$∆{XY}_{dc}\_z$	Mean distance between each sample and the centroid
$∆{XY}_{\theta }\_z$	Mean angle formed by in one vertex by the centroid
$∆{XY}_{\beta }\_z$	Mean angle formed by in the centroid by two vertices
$∆{XY}_{\theta \beta }\_z$	Mean difference between $∆{XY}_{\theta }\_z$ and $∆{XY}_{\beta }\_z$
$∆{XY}_{\mathrm{pr}}\_z$	Mean number of points inside of regions formed by two vertices and the centroid
$∆{XY}_{A_{r}}\_z$	Mean area of regions formed by two vertices and the centroid

X and Y refers to either the BBI or TT time series; z refers to incremental ( up ), no change ( nc ) or decremental activity ( d ).

## 5 Classification and validation

Support vector machines (SVM) method is useful for classification tasks where the classes are not linearly separable in the original space. By transforming the data into a higher dimensional space, SVM can transform a complex non-linear problem into a simple linear one. This is achieved through the optimization of a hyperplane defined by the SVM function, being $X=\left\{x_{1},\ldots ,x_{L}\right\}\forall R$ for a given set of data vectors and $Y=\left\{y_{1},\ldots ,y_{L}\right\}\forall R$ their corresponding labels:

$$f\left(x\right)=wz+b=\sum_{i}^{L}\alpha_{i}y_{i}K\left(x_{i}y_{i}\right)+b$$ (4)

where $K\left(x_{i}y_{i}\right)$ is known as the kernel function that shapes the hyperplane and $\alpha_{i}$ and b defines the efficiency of the classifier under optimal conditions. From all the possible kernel types we evaluated the Gaussian, Laplace and ANOVA kernels.

The Gaussian kernel is often used to model radially distributed data and is given by,

$$K\left(x,y\right)=e^{\left({\left\|x-y\right\|}^{2}/2\sigma^{2}\right)}$$ (5)

where σ is a penalization term.

The Laplace kernel is a less σ influenced version of the Gaussian kernel and expressed as:

$$K\left(x,y\right)=e^{\left({\left\|x-y\right\|}^{2}/2\sigma \right)}$$ (6)

where σ is a penalization term.

The ANOVA kernel is used on multidimensional support vector regression models and given by,

$$K\left(x,y\right)=\sum_{k=1}^{n}e^{{\left.{\left(-\sigma \left(x^{k}-y^{k}\right)\right.}^{2}\right)}^{d}}$$ (7)

where σ and d are optimization indices.

The classification problem is then solved by maximizing the margin while minimizing the training error. Using the Lagrange multipliers method, a dual formulation is obtained:

$$minP\left(w,b\right)=\frac{1}{2}{\left\|w_{m}z\right\|}^{2}+C\sum_{i}K_{i}\left[y_{i}f\left(x_{i}\right)\right]$$ (8)

where C is a penalty parameter. Despite C having no direct meaning, when its value increases, the penalty assigned to errors is stronger, narrowing the decision boundary.

Each feature was scaled and normalized (zero mean and unit variance) in order to avoid scaling biases. For each iteration of features, the model was built by optimizing the value of C for each of the kernels considered, by iterating different values of σ and d . The indices that showed statistical differences and low correlation were used in pairs to build several SVM models.

A Mann-Whitney non-parametric statistical test was applied to evaluate the statistically significant differences between all the indices. Any index with a *p*-value ≤ 0.05 was considered statistically significant. The leave-one-out cross-validation method was used to validate the results. The classification results are presented in terms of accuracy $\left(Acc\right)$ , sensitivity $\left(Sn\right)$ , and specificity $\left(Sp\right)$ .

## 6 Results and discussion

This work aimed to characterize the cardiovascular and cardiorespiratory activity of patients with ischemic or dilated cardiomyopathy. A total of 168 indices were obtained during the characterization process. The results were analyzed considering the following comparisons:

- Ischemic *vs*. dilated cardiomyopathy patients (ICM *vs*. DCM)
- Cardiomyopathy patients *vs*. control subjects (CMP *vs*. CON)
- Ischemic cardiomyopathy patients *vs*. control subjects (ICM *vs*. CON)
- Dilated cardiomyopathy patients *vs*. control subjects (DCM *vs*. CON)

**FIGURE 3:**
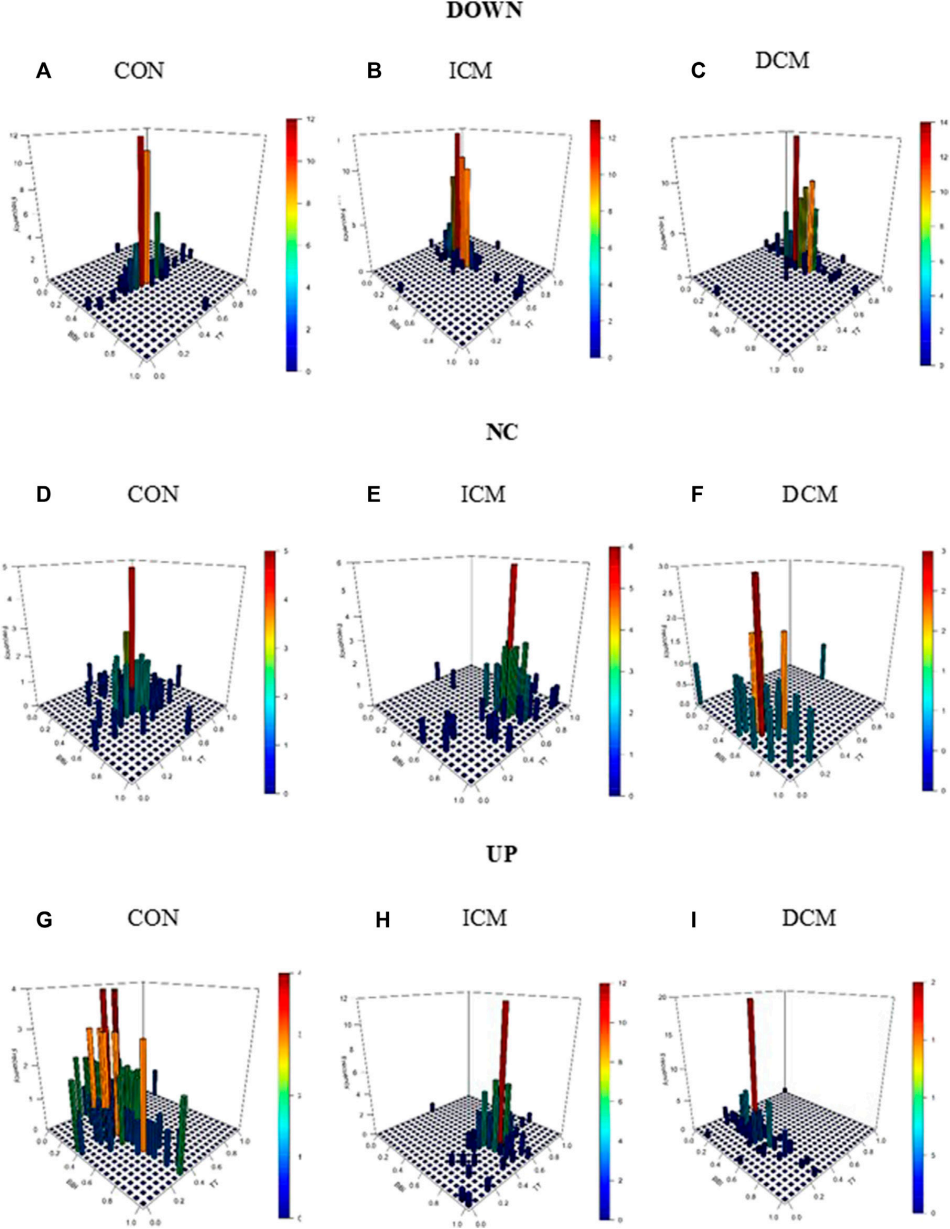
Statistical characterization of cardiorespiratory activity based on the sub-space for systolic blood pressure activity: down for **(A)** CON subject, **(B)** ICM and **(C)** DCM patients; no-change for **(D)** CON subject, **(E)** ICM and **(F)** DCM patients; and up for **(G)** CON subject, **(H)** ICM and **(I)** DCM patients.

**FIGURE 4:**
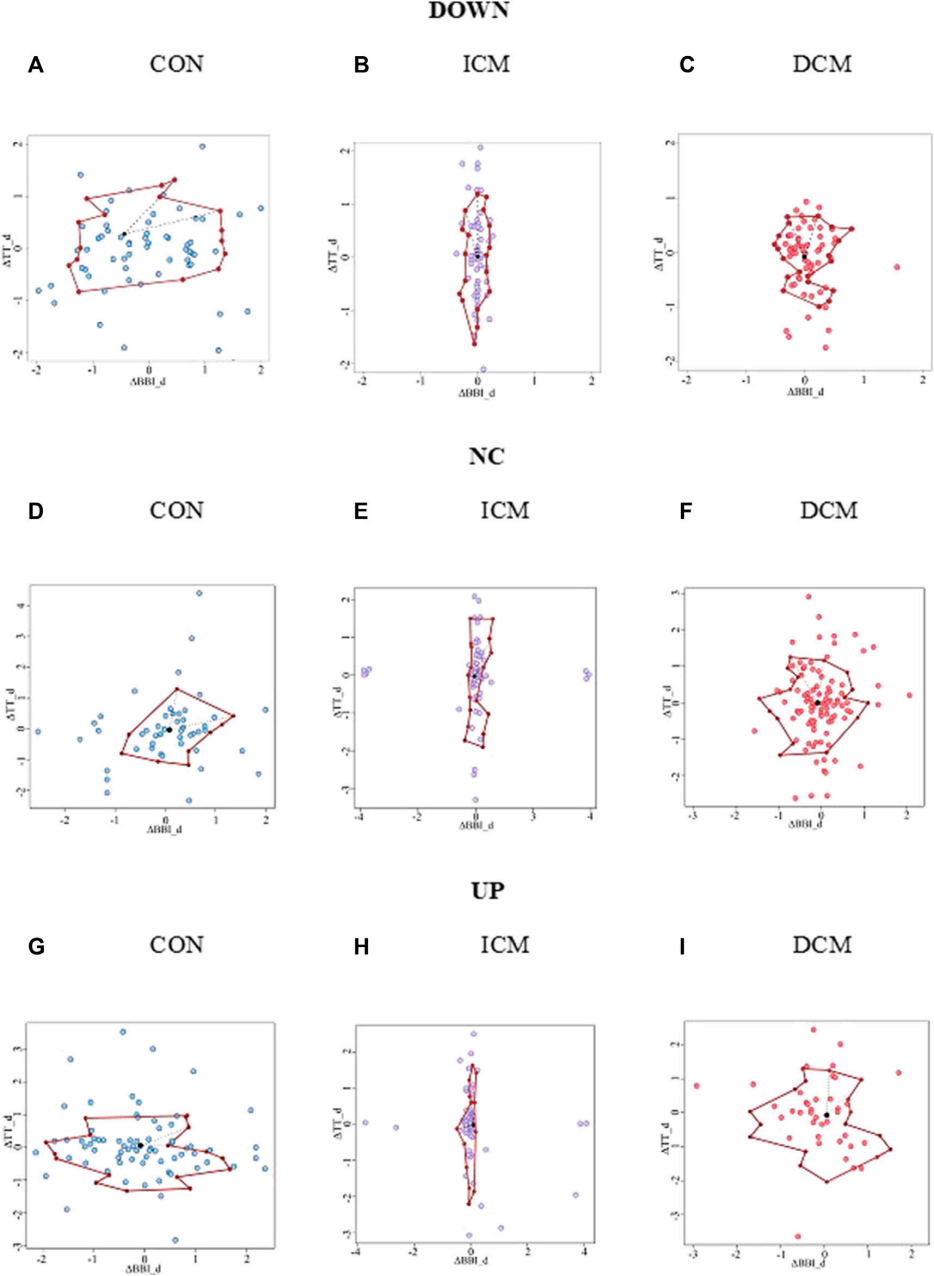
Morphological characterization of cardiorespiratory activity based on the sub-space for systolic blood pressure activity: down for **(A)** CON subject, **(B)** ICM and **(C)** DCM patients; no-change for **(D)** CON subject, **(E)** ICM and **(F)** DCM patients; and up for **(G)** CON subject, **(H)** ICM and **(I)** DCM patients.

Figure 3 provides an example of the statistical characterization of cardiorespiratory activity based on systolic blood pressure activity according to the different sub-spaces: down (a, b, c), no-change (d, e, f), and up (g, h, i) for a CON subject (a, d, g), an ICM patient (b, e, h), and a DCM patient (c, f, i). Figure 4 illustrates an example of the morphological characterization of cardiorespiratory activity based on systolic blood pressure activity according to the different sub-spaces: down (a, b, c), no-change (d, e, f), and up (g, h, i) for a CON subject (a, d, g), an ICM patient (b, e, h), and a DCM patient (c, f, i).

In the comparison of ICM and DCM patients, 9 indices presented statistically significant differences, and relatively low correlation. For the CMP patients vs. CON group, 13 indices presented statistical differences. When each pathology was compared to the CON group, 10 indices presented differences when compared to ICM patients, and 19 when compared to DCM patients. These indices were used to build different SVM models, determining the best classifiers in each case. Table 3 presents the most relevant indices for each comparison in terms of mean value and standard deviation.

**TABLE 3:** Significant indices for ICM *vs*. DCM, CMP *vs*. CON, ICM *vs*. CON, and DCM *vs*. CON comparisons, presented in terms of mean value and standard deviation.

ICM *vs*. DCM			
Index (nu)	ICM (24)	DCM (17)	*p*-value
SBP - ${∆TT}_{m}$ -d	0.007 ± 0.12	−0.07 ± 0.07	0.02
SBP - ${∆TT}_{sd}$ -u	1.00 ± 0.17	0.89 ± 0.14	0.02
SBP *-* ${∆BBI}_{sd}$ *-nc*	0.85 ± 0.28	1.09 ± 0.18	0.009
SBP *-* $∆{BBI\_TT}_{\theta }$ *-u*	76.25 ± 29.47	49.23 ± 39.59	0.01
SBP - ${∆TT}_{CV}$ -d	−3.15 ± 31.10	−31.2 ± 52.2	0.002
DBP - ${∆TT}_{CV}$ -d	1.05 ± 0.15	0.96 ± 0.14	0.03

*CMP vs. CON*			
Index (nu)	CMP (41)	CON (39)	*p*-value
DBP - ${∆BBI}_{p}$ -u	61.60 ± 18.73	87.43 ± 13.18	<0.00001
DBP *-* ${∆BBI\_TT}_{Av}$ *-u*	0.17 ± 0.15	0.27 ± 0.09	0.004
SBP - ${∆BBI}_{IQR}$ *-u*	0.66 ± 0.53	1.09 ± 0.45	0.00006
SBP - ${∆BBI}_{IQR}$ *-nc*	0.60 ± 0.47	1.04 ± 0.43	0.00007
SBP - ${∆BBI}_{IQR}$ *-d*	0.62 ± 0.48	1.03 ± 0.45	0.0003

*ICM vs. CON*			
Index (nu)	ICM (24)	CON (39)	*p*-value
SBP - ${∆BBI}_{K}$ *-nc*	14.70 ± 24.17	4.19 ± 8.55	0.01
DBP *-* ${∆BBI}_{p}$ *-u*	61 ± 15.16	87.43 ± 13.18	<0.00001
SBP - ${∆BBI}_{IQR}$ *-d*	0.74 ± 0.57	1.09 ± 0.45	0.004
SBP - ${∆TT}_{CV}$ *-d*	−3.15 ± 31.10	−8.43 ± 54.18	n.s

*DCM vs. CON*			
Index (nu)	DCM (17)	CON (39)	*p*-value
SBP *-* ${∆TT}_{IQR}$ *-u*	0.63 ± 0.20	0.75 ± 0.21	0.03
SBP *-* ${∆BBI\_TT}_{A}$ *-u*	1.78 ± 1.73	4.02 ± 1.80	0.00009
SBP - ${∆BBI}_{IQR}$ *-d*	0.54 ± 0.46	1.09 ± 0.45	0.0001
SBP - ${∆TT}_{CV}$ *-d*	−31.2 ± 52.2	−8.43 ± 54.18	0.006

*nu: normalized units.

**FIGURE 5:**
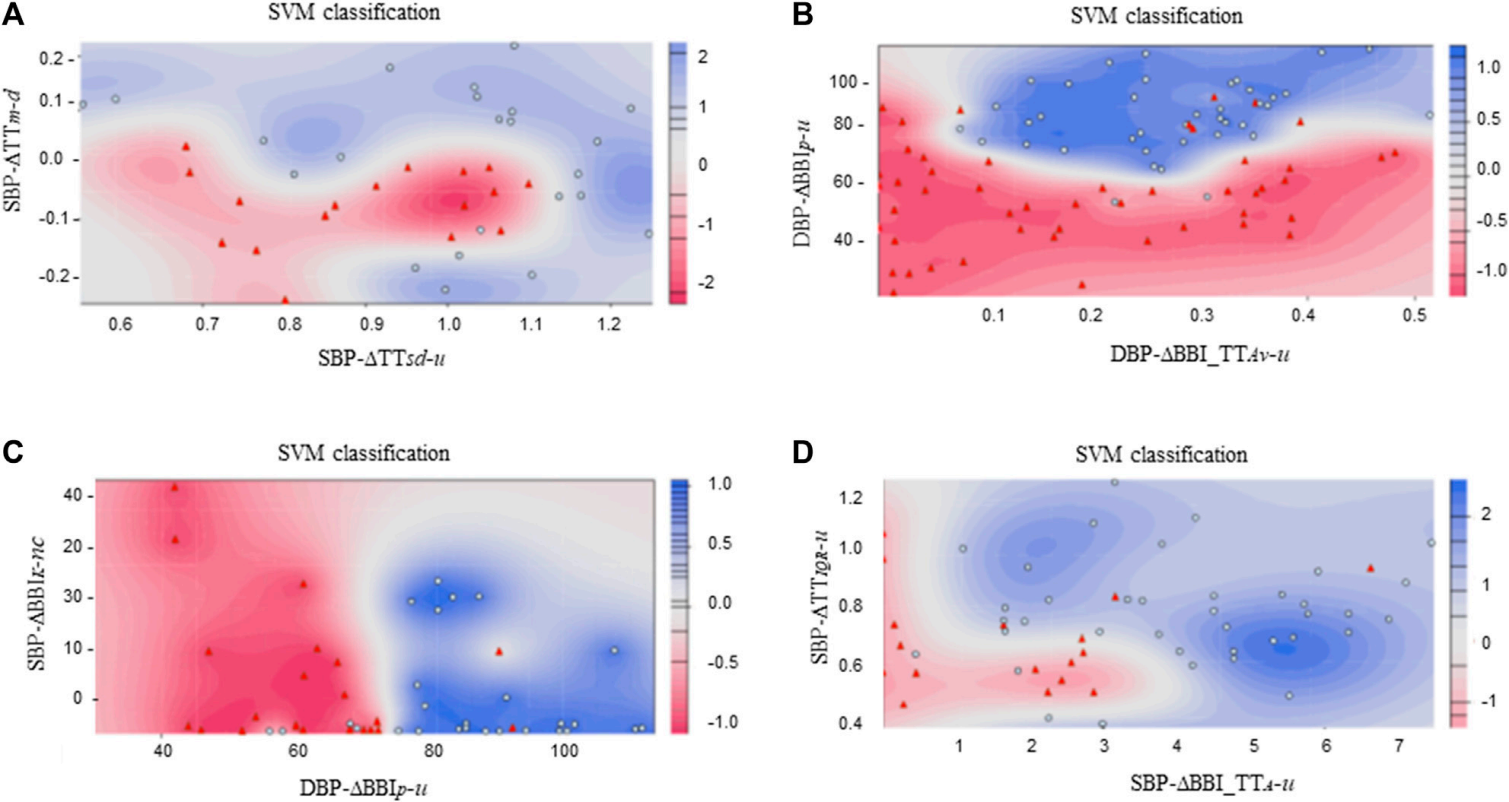
SVM classification plots considering: **(A)** ICM (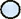) *vs*. DCM (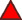) patients, **(B)** CMP (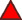) patients *vs*. CON (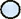) subjects, **(C)** ICM (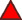) patients *vs*. CON (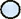) subjects, and **(D)** DCM (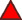) patients *vs*. CON (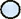) subjects.

The SBP - ${∆TT}_{m}$ *-d* and SBP - ${∆TT}_{sd}$ *-u* were the indices used to build the optimal SVM model for the ICM vs. DCM comparison, achieving 92.7% accuracy, 94.1% sensitivity and 91.7% specificity. The optimal model for the CMP *vs*. CON comparison was built with the DBP - ${∆BBI}_{p}$ *-u* and DBP - ${∆BBI\_TT}_{A_{v}}$ *-u*, obtaining 86.2% accuracy, 82.9% sensitivity, and 89.7% specificity. The indices for the optimal ICM *vs*. CON model were the SBP - ${∆BBI}_{K}$ *-nc* and DBP - ${∆BBI}_{p}$ *-u*, achieving 88.9% accuracy, 87.5% sensitivity and 89.7% specificity. The SBP - ${∆TT}_{IQR}$ *-u* and SBP - ${∆BBI\_TT}_{A}$ *-u* indices were used to build the SVM model for DCM vs. CON, obtaining 87.5% accuracy, 76.5% sensitivity and 92.3% specificity. The Gaussian kernel was used in both the ICM vs. DCM and the DCM vs. CON SVM models; the Laplace kernel was used in the CMP vs. CON and the ICM vs. CON comparisons. The classification results and the SVM scoreplot are shown in Table 4; Figure 5, respectively.

**TABLE 4:** Accuracy (Acc), sensitivity (Sn), and specificity (Sp), obtained with the best SVM model for each classification group.

Groups	*C*	*σ*	*Acc(%)*	*Sn(%)*	*Sp(%)*
ICM *vs*. DCM	2.2	1.5	92.7	94.1	91.7
CMP *vs*. CON	1	1.5	86.2	82.9	89.7
ICM *vs*. CON	1	2	88.9	87.5	89.7
DCM *vs*. CON	5	0.8	87.5	76.5	92.3

The interaction between cardiorespiratory and vascular activity was explored and characterized to analyze the behavior of these systems in patients diagnosed with ischemic or dilated cardiomyopathy. Afterwards, the best indices were used to classify these patients, through the introduction of new information about the response of the cardiac and respiratory systems, based on vascular activity.

Our results indicate a decreasing respiratory response in DCM patients when systolic blood pressure activity decreases ( SBP - ${∆TT}_{m}$ -d), in contrast to the more stable response in ICM patients. In addition, ICM patients exhibited higher variance in respiratory activity a as response to decreased systolic or diastolic blood pressure activity ( SBP - ${∆TT}_{CV}$ -d, DBP - ${∆TT}_{CV}$ -d).

The decreased respiratory activity observed during drops in systolic blood pressure in DCM patients suggests a faster breathing response to decreasing blood pressure. Changes in breathing rhythm are known to affect blood pressure levels (Nuckowska et al., 2019). In addition, several respiratory abnormalities have been associated before with left ventricular impairment (Mortara et al., 1999). On the other hand, it have been stated before that respiratory performance may be dependent on cardiac function, especially in heart failure patients (Nishimura et al., 1994). We hypothesize that the respiratory activity in DCM patients compensates for the impaired cardiac-dependent regulation of blood pressure during the baroreflex response.

The comparison of SBP activity when the values correspond to “no change” revealed that cardiac response was more stable in ICM patients ( SBP - ${∆BBI}_{sd}$ -nc), than in DCM patients. This may be a sign of healthier autonomic regulation in ICM patients, who have retained some parasympathetic responsiveness (Malfatto et al., 2001). Some studies have reported that DCM patients experience impairment of cardiac autonomic regulation (Yi et al., 1997; Momose et al., 2001). Therefore, this impairment may explain the differences observed in the cardiac behavior of DCM patients vs. ICM patients in contrast to the ischemic-related etiology in ICM patients (Malfatto et al., 2001; Cuponne et al., 2012).

In the presence of increased SBP activity, the respiratory response is more disperse in ICM patients than in DCM patients ( SBP - ${∆TT}_{sd}$ -u). These differences could be attributed to stimulation of the vagal pulmonary mechanoreceptors, which, mechanical in origin, are not influenced by ischemic stimulus (Lombardi et al., 1984). In addition, changes in respiratory patterns has shown to induce changes in blood pressure behavior (van de Borne et al., 1998). As increased respiratory variability has been observed in elderly people in a previous work (Kaplan et al., 1991), we suggest that the differences found in DCM patients are due to a more pronounced impairment of their autonomous regulation.

In addition, in cases of increased in SBP activity, the mean angle formed by each vertex and the centroid of its fitting cloud of points associated with cardiorespiratory activity ( SBP - ${∆BBI\_TT}_{ϴ}$ -u) had higher values in ICM patients, while the values of DCM patients were more scattered. These patterns suggest that ICM patients showed more stable cardiorespiratory activity than DCM patients.

An analysis of overall cardiac behavior among CMP patients, in general showed a reduced response to increasing DBP activity ( DBP - ${∆BBI}_{p}$ -u) compared to CON subjects. The differences found in the DBP - ${∆BBI\_TT}_{A_{v}}$ *-u* indicated lower cardiac and respiratory variability in CMP patients when DBP activity increases. The interquartile range of the cardiac activity of CMP patients across every SBP pattern considered ( SBP - ${∆BBI}_{IQR}$ -u, SBP - ${∆BBI}_{IQR}$ -nc, SBP - ${∆BBI}_{IQR}$ -d) presented lower values when compared to the subjects of the CON group. The results of these indices indicate lower heart rate variability in patients with cardiomyopathies. Lower cardiac and respiratory variabilities are typically associated with a cardiovascular dysfunction (Lombardi et al., 1984; Kaplan et al., 1991; Claria et al., 2001). We hypothesize that the cardiac and respiratory systems in CMP patients have a more limited ability to regulate incremental blood pressure changes compared to CON subjects. This limitation may contribute to the prevalence of hypertension in these type of patients (Kuroda et al., 2015).

A similar behavior was observed for increased DBP activity, when ICM patients were compared to the CON subjects ( DBP - ${∆BBI}_{p}$ -u), and the cardiac activity was lower in ICM patients. The overall observations regarding the interquartile range of the cardiac activity for decreasing blood pressure were also present in the ICM vs. CON ( SBP - ${∆BBI}_{IQR}$ -d) and DCM *vs*. CON ( SBP - ${∆BBI}_{IQR}$ -d) comparisons. These indices suggest that cardiac activity is diminished in both ICM and DCM patients when compared with the CON subjects.

Cardiac and respiratory variability differences were also found when the DCM patients were compared to the CON subjects for increased SBP activity ( SBP - ${∆BBI\_TT}_{A}$ -u), and lower values were recorded in DCM patients. It have been stated before that DCM is associated with loss in heart rate dynamics in relation to blood pressure regulation (Baumert et al., 2005). A similar behavior have being modelled before through non-linear methods (Mohammed et al., 2018). In addition, an influence of an impaired myocardial performance in the behavior of the cardiovascular activity have being hypothesized before (Voss et al., 2006). We found no significant differences between respiratory variance ( SBP - ${∆TT}_{CV}$ -d) when comparing ICM patients and CON subjects for decreasing blood pressure activity. In contrast, differences were recorded in respiratory variance between DCM patients and CON subjects. These results suggest specific patterns among DCM patients in their respiratory response to decreasing blood pressure activity. These observations are consistent with an earlier study in which the respiratory sinus arrhythmia values were found to be lower in ICM and DCM patients in comparison with controls (Giraldo et al., 2018).

This study has some limitations that should be taken into consideration. One of them is the age disparity between the control group and the patient group. However, the influence of age on the study of cardiac activity variability has been extensively studied in previous works (Porta et al., 2014; Voss et al., 2015). Nonetheless, this limitation does not affect our results when comparing variations among the different parameters that describe cardiorespiratory and vascular relationships, thereby mitigating possible biases derived from the passing of time.

Another limitation is related to the comorbidities and confounding factors presented by the patients, which may influence the autonomic regulation of the systems. Nevertheless, this study allows independent quantification of cardiorespiratory interactions as a function of vascular activity. Assessing the relative variations between these systems can contribute to the early analysis of the evolution of the patients.

## 7 Conclusion

In conclusion, the analysis of the interaction between cardiac and respiratory activity and blood pressure provides novel insight into the classification of patients with this type of disease. We found respiratory patterns that are more prevalent in dilated cardiomyopathy patients. In general, cardiac and respiratory variability showed lower values in these patients compared to the patterns observed in healthy subjects. Furthermore, these results suggest more specific patterns in the respiratory response with decreased blood pressure activity for patients with dilated cardiomyopathy.

The work presented here may provide physicians with new symptom-independent information about these types of cardiomyopathies, which may help improving the diagnosis of these patients. This method not only serves to analyze the behavior of ischemic and dilated cardiomyopathy patients, but also introduces a new procedure with which to analyze the dynamical behavior between other coupled systems. However, additional features with clinical information about the patients should be considered to increase the discrimination between the groups. This study is limited by the size of the dataset employed and, consequently, the results discussed in this work are of an exploratory nature and should be validated using a larger dataset.

## Data Availability

The data analyzed in this study is subject to the following licenses/restrictions: Data supporting the findings of this study are available from BG, but restrictions apply to the availability of these data, which were used under license for the current study, by what are not publicly available. The data set is not publicly available due to the conditions that were established when the protocol was defined and the patients were registered. Requests to access these datasets should be directed to BG, Beatriz.Giraldo@upc.edu.
